# Genomic Insight into the Mobility of Antibiotic Resistance Genes in Multidrug-Resistant *Escherichia coli* Isolated from Dewatered Sludge Cakes

**DOI:** 10.3390/antibiotics15040364

**Published:** 2026-04-01

**Authors:** Taeun Kim, Yeojin Han, Seohyeon Je, Minwoo Kim, Hokyung Song

**Affiliations:** Department of Environmental Engineering, Chosun University, Chosundae 60 5-gil, Dong-gu, Gwangju 61452, Republic of Korea

**Keywords:** antibiotic resistance gene, mobile genetic element, multidrug resistance, *Escherichia coli*, dewatered sludge cake, wastewater treatment plant, whole genome sequencing, horizontal gene transfer

## Abstract

**Background/Objectives**: Municipal wastewater treatment plants (WWTPs) act as reservoirs for antibiotic-resistant bacteria, which pose a threat to global public health. In this study, we used whole-genome sequencing (WGS) to characterize antibiotic resistance genes (ARGs) and their association with mobile genetic elements (MGEs) in five multidrug-resistant (MDR) *Escherichia coli* isolates from dewatered sludge cake samples collected from a municipal WWTP in Cheongju, Republic of Korea. **Methods**: Susceptibility to nine antibiotics was evaluated via disk diffusion assay. Among the isolates exhibiting multidrug resistance (MDR) to three or more antibiotic classes, five isolates were randomly selected for whole-genome sequencing using the Illumina NovaSeqX platform. Additionally, we compared the genomic structures of five WWTP isolates with 35 environmental *E. coli* isolates from South Korea deposited in the NCBI pathogen database. ARGs and MGEs, including plasmids, integrons, and insertion sequences (ISs), were detected in the genome assemblies. **Results**: ARGs were differentially distributed between chromosomal and plasmid-derived contigs. Efflux pump-related genes were predominantly located on the chromosome across all isolates, whereas several beta-lactamase genes (e.g., *bla*_TEM-30_ and *bla*_TEM-33_), fluoroquinolone, and tetracycline resistance genes were localized on putative plasmid contigs. Furthermore, we characterized specific MGEs associated with these ARGs, including a class 1 integron gene cassette (*dfrA17–aadA5–qacEΔ1–sul1*) and an IS-mediated module (*mph(A)–mrx–IS6100*). Core-genome multilocus sequence typing (cgMLST) revealed that these MDR isolates represented diverse genetic lineages rather than a single clonal cluster. **Conclusions**: The results from this study highlight the necessity of enhanced post-treatment management of wastewater byproducts and WGS-based surveillance to mitigate the environmental spread of MDR bacteria.

## 1. Introduction

Antibiotics have been used for several decades to prevent and treat bacterial infections in animals, including humans. However, due to the overuse and misuse of antibiotics, antimicrobial resistance (AMR) has emerged as a serious public health crisis [1]. The World Health Organization has classified AMR as one of the most serious public health threats. Globally, approximately 1.27 million people died of AMR in 2019 [2]. Annual deaths due to AMR are estimated to reach 10 million by 2050 if no management actions are taken [3].

AMR is not restricted to the clinical sector because antibiotic-resistant bacteria (ARB) and antibiotic resistance genes (ARGs) can spread throughout diverse environments. Antibiotics cannot be completely degraded in the human body; up to 90% of antibiotics are excreted through urine or feces [4,5,6] and enter wastewater treatment plants (WWTPs). Although wastewater passes through biological and physicochemical treatment processes before final release, ARB and ARGs often remain in the effluents and are released into the aquatic environments [7,8,9]. Lee et al. (2023) showed that ARGs such as *aadA*, *sul1* and class A beta-lactamase were significantly more abundant downstream than upstream of WWTPs [10]. Similarly, Shin et al. (2023) reported a higher abundance of ARGs such as *aadA2*, *aadA*, and *aadA5* in WWTP effluents than in natural stream environments [11]. These results suggest that WWTPs can accumulate ARB and ARGs, which can then spread to natural freshwater environments.

Activated sludge treatment is one of the most popular wastewater treatment methods worldwide. Although this method is efficient for treating urban wastewater, which includes high concentrations of organic compounds, a large amount of sludge is produced as a byproduct [12]. Sludge is generally released as dewatered sludge cakes via concentration, anaerobic digestion, and dehydration [13]. During sludge digestion, high microbial loads and organic matter may facilitate ARG accumulation in the final dewatered sludge cake [14,15,16,17].

In 2024, approximately 4,458,053 tons of solid waste was produced from 4469 WWTPs in South Korea, and approximately 27.9% of processed sludge waste was reused through composting processes [18]. Although reusing sludge through composting is beneficial for reducing waste and recovering resources, remaining ARGs may enter the ecosystem through compost or soil amendments and spread via horizontal gene transfer (HGT). It may subsequently spread through soil and agricultural water and eventually threaten the health of animals, including humans [19,20,21,22].

Previous studies on ARGs in WWTPs have focused on comparing ARG concentrations in influents and effluents to assess treatment efficacy [23,24] and have mainly concentrated on liquid samples [25]. In many cases, methods such as quantitative polymerase chain reaction (qPCR) or high-throughput qPCR (HT-qPCR), which identify defined targets, have been used for ARG monitoring [9,23,26]. Using these methods, non-target ARGs and ARGs originating from point mutations cannot be detected. In addition, identifying genomic associations between ARGs and mobile genetic elements (MGEs) is difficult, which limits the assessment of the potential mobility of ARGs [27].

With the advancement and reduced cost of next-generation sequencing technologies, whole-genome sequencing (WGS) has been widely applied across various fields. WGS enables bacterial identification at the strain level and can be applied to track the potential transmission routes of ARBs through comparative genomics [28,29]. For example, Mbanga et al. (2021) used WGS-based analysis of multidrug-resistant (MDR) *E. coli* isolated from effluents and adjacent stream environments of WWTPs and reported that a large proportion of the identified ARGs were located on plasmids or close to MGEs, such as class 1 integrons, ISs, and transposons [30].

In this study, we used WGS to characterize the antibiotic resistance genomic profiles of *E. coli* strains isolated from dewatered sludge cakes of WWTP located in Cheongju, South Korea, to identify ARGs and their association with MGEs, and to assess the potential mobility of ARGs through HGT.

## 2. Results

### 2.1. Antibiotic Susceptibility Test Based on Disk Diffusion Assay

**Figure 1:**
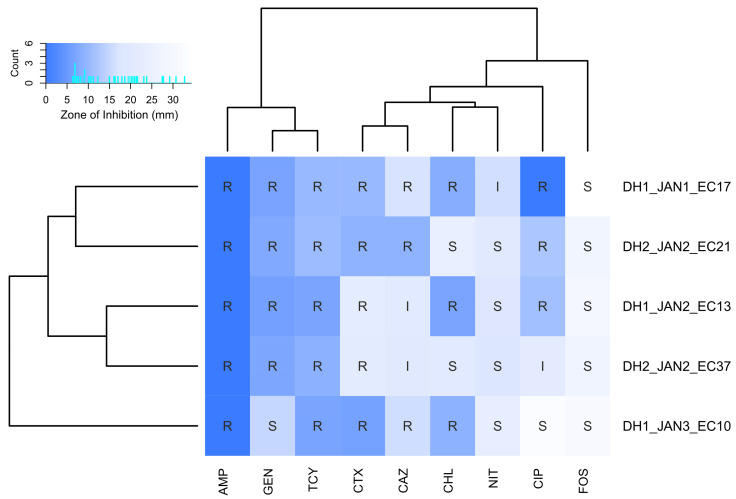
Antibiotic susceptibility profiles of five *Escherichia coli* strains determined using the disk diffusion method. Heatmap colors represent inhibition zone diameters, whereas overlaid text indicates categorical interpretation: resistant (R), intermediate (I), or susceptible (S). AMP: ampicillin; GEN: gentamicin; TCY: tetracycline; CTX: cefotaxime; CAZ: ceftazidime; CHL: chloramphenicol; NIT: nitrofurantoin; CIP: ciprofloxacin; FOS: fosfomycin.

The disk diffusion assay of the five *E. coli* isolates from the WWTP showed that all five isolates were resistant to ampicillin, cefotaxime, and tetracycline (Figure 1). Three isolates were resistant to ceftazidime and two isolates showed an intermediate phenotype against ceftazidime. Four isolates were resistant to gentamicin, and three isolates were resistant to chloramphenicol. Three isolates were resistant to ciprofloxacin, one showed intermediate resistance, and the other was susceptible. Four isolates were susceptible to nitrofurantoin and one isolate showed intermediate resistance. All isolates were susceptible to fosfomycin.

### 2.2. Genome Assembly and Taxonomic Annotation

The BUSCO analysis demonstrated the high quality of the assembled genomes; all five isolates exhibited a completeness of 99.1%, with no fragmented genes and only 0.9% missing genes. Four of the isolates had 99.1% single-copy genes and zero duplicated genes, whereas DH2_JAN2_EC21 had 98.3% single-copy genes and 0.9% (one gene) duplicated genes. GTDB-tk results showed that all five isolates belonged to *E. coli*, and the average nucleotide identity with the closest reference genome was 96.33–97.08%.

### 2.3. Diversity and Distribution of ARGs

**Figure 2:**
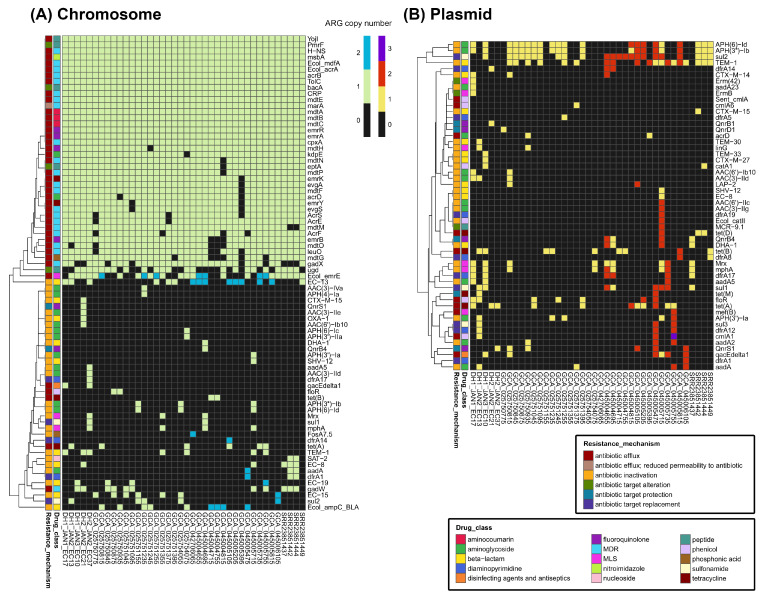
Distribution and copy numbers of antibiotic resistance genes (ARGs) across 40 *Escherichia coli* strains (5 isolates from dehydrated sludge cakes and 35 environmental reference strains). (**A**) Heatmap displaying ARGs located on the chromosomal contigs. (**B**) Heatmap displaying ARGs located on plasmid contigs.

The genomic localization (chromosome vs. plasmid) of ARGs was compared between five WWTP-oriented isolates and 35 environmental reference strains (Figure 2). Statistical analysis revealed that the abundance of genes conferring resistance to disinfecting agents and antiseptics on the chromosome was significantly higher in WWTP-derived strains than in reference isolates (adjusted *p* = 0.003; Table S1). In contrast, no significant differences were observed for other drug classes across any genomic localizations.

Several resistance-related elements were identified in the chromosomal contigs, including efflux pump genes (*acrAB–tolC*, *mdtABC*, *mdfA*, *yojI*, and *msbA*) and regulatory genes (*marA*, *crp*, and *cpxA*). Among the beta-lactam resistance genes, *bla*_TEM-1_ was found on both chromosomes and plasmids in several strains. DH2_JAN2_EC21 harbored *bla*_OXA-1_ and *bla*_CTX-M-15_, whereas several reference strains of environmental origin harbored *Ecol_ampC_BLA* in their chromosomal contigs. DH1_JAN2_EC13 harbored *bla*_TEM-30_ and DH1_JAN3_EC10 harbored *bla*_TEM-33_ on plasmid contigs, whereas the reference strain SRR23851442 harbored *bla*_TEM-1_ and *bla*_CTX-M-15_ on its plasmid contigs. *bla*_DHA-1_ and *bla*_SHV-12_ genes were found only in the reference strains and were distributed on both chromosomes and plasmids.

Notably, certain plasmid-mediated quinolone resistance (PMQR) determinants were identified within the bacterial chromosomal contigs rather than on plasmid contigs. For example, in DH2_JAN2_EC21, *qnrS1* was located on a chromosomal contig, whereas *qnrB1* was located on a plasmid contig. DH1_JAN1_EC17 and DH1_JAN2_EC13 lacked *qnr* genes. For tetracycline resistance genes, *tet(A)* and *tet(B)* were found in both WWTP and environmental strains.

*qacEΔ1*, a disinfectant resistance gene, was found on plasmid contigs in six strains and on chromosomal contigs in two strains. Among them, five strains harbored *sul1* and five strains harbored *sul2*. Strains containing *qacEΔ1* frequently had aminoglycoside and diaminopyrimidine resistance genes, although their combinations and localizations differentiated between strains. For example, DH1_JAN1_EC17, DH1_JAN3_EC10, DH2_JAN2_EC37, and GCA_045005735 had *aadA5* and *dfrA17*. Among them, DH2_JAN2_EC37 had *qacEΔ1*, *sul1*, *aadA5*, and *dfrA17* on its chromosomal contigs, whereas the same gene combination was found on plasmid contigs in DH1_JAN3_EC10 and GCA_045005735.

### 2.4. Antibiotic Resistance Phenotype and Genotype Comparison and Variant Analysis

When the antibiotic resistance phenotypes (determined by disk diffusion assay) were compared with the genotypes (determined by the Comprehensive Antibiotic Resistance Database [CARD] Protein Homolog Model annotation), most antibiotics—including ampicillin, cefotaxime, and tetracycline—showed high levels of consistency. However, several mismatches were observed for ciprofloxacin.

To further investigate these discrepancies, an analysis based on the CARD Protein Variant Model was performed. Comparison with the CARD variant model reference sequences revealed one or more amino acid substitutions in a total of 16 genes relative to the wild-type reference sequences (Table S2). When these variants were compared against the catalog of resistance-associated substitutions provided by CARD, only a subset of the mutations matched the known resistance markers (Table S2).

Specifically, in strains DH1_JAN1_EC17 and DH1_JAN2_EC13, which exhibited a ciprofloxacin-resistant phenotype in the disk diffusion assay but lacked plasmid-mediated quinolone resistance (PMQR) genes (e.g., *qnrS1*, *qnrA*) [31] in the homolog model, mutations in the quinolone resistance-determining region (QRDR) were observed [32,33] (Table S2). Sequence alignment with *E. coli* K-12 MG1655 confirmed S83L and D87N substitutions in GyrA, as well as an S80I substitution in ParC in both strains (Figure S1).

Regarding fosfomycin, all five WWTP-derived strains exhibited a susceptible phenotype in the disk diffusion assay, and no plasmid-mediated fosfomycin resistance genes (e.g., *fosA*, *fosB*) were detected in the homolog model. However, several protein sequence variants related to *ptsI*, *uhpT*, *cyaA*, and *uhpA* were identified (Table S2). Among these, the E350Q substitution in UhpT (Ecol_UhpT_FOF), a mutation known to confer fosfomycin resistance, was identified in strains DH1_JAN1_EC17, DH1_JAN3_EC10, and DH2_JAN2_EC21. Additionally, the S352T substitution in CyaA (Ecol_cyaA_FOF) was found in strain DH1_JAN3_EC10 (Table S2).

### 2.5. Sequence Types (STs) and cgMLST-Based Phylogenetic Analysis

**Figure 3:**
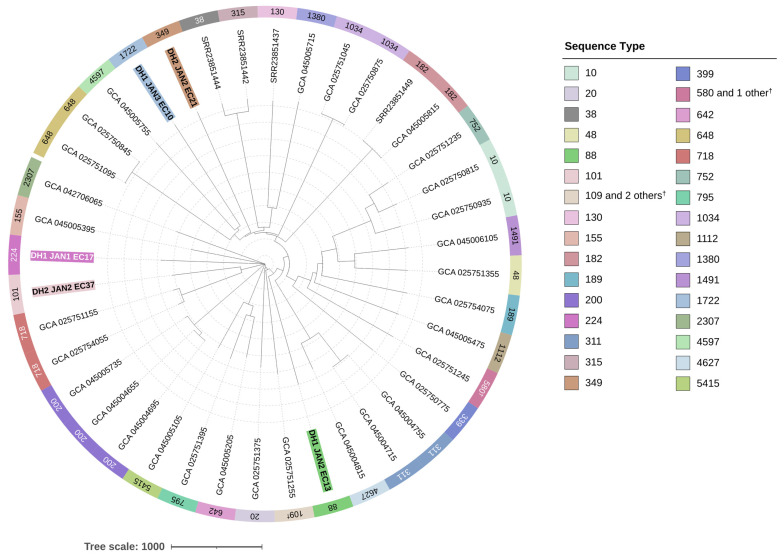
Core-genome MLST-based phylogenetic tree of 40 *Escherichia coli* strains. The tree includes five isolates obtained from the dewatered sludge (DH1, DH2) of two Cheongju wastewater treatment plants (highlighted with bold text and distinct background colors), together with 35 reference strains selected from the NCBI database. For some isolates, STs could not be confidently assigned because one or more loci did not match any known alleles in the PubMLST database; such cases are indicated with notations like “109 and 2 others ^†^” or “580 and 1 other ^†^” to reflect this uncertainty.

The STs of 38 out of 40 isolates were determined (95.0%) by MLST analysis, and among the 38 isolates, 30 different STs were identified (Figure 3). Three isolates belonged to ST200, and two isolates each belonged to ST10, ST182, ST311, ST648, ST718, and ST1034. The strain types of the five WWTP isolates were different (ST88, ST101, ST224, ST349, and ST1722), and no overlapping STs were found in the reference isolates of environmental origin. In the phylogenetic tree constructed based on the cgMLST results, the WWTP strains did not cluster with each other but were rather dispersed (Figure 3).

### 2.6. MGEs Associated with ARGs

Using MOB-suite and PlasmidFinder, 1034 plasmid contigs were identified from 40 isolates; of these, 518 were classified as conjugative plasmids, 182 as mobilizable, and 334 as non-mobilizable. In the five WWTP isolates, 84 contigs were identified as plasmid contigs, of which 36.9% were conjugative, 10.7% were mobilizable, and 52.4% were non-mobilizable. In the WWTP isolates, IncF, IncI, and IncQ plasmids and Col replicons were identified (Table S3). Several contained ARGs; for example, in DH1_JAN3_EC10, *aph(3″)-Ib*, *aph(6)-Id*, and *sul2* were identified on the IncQ1 plasmid contig, and in DH2_JAN2_EC37, *tet(A)* and *qnrS1* were identified on the IncFII contig. In contrast, in 35 isolates of environmental origin, IncI, IncB/O/K/Z, IncX, IncR, IncC, IncH, and various Col-type replicons were observed. Additionally, among 121 contigs, 14 (11.6%) carried antibiotic resistance genes (ARGs), including *blaTEM-1*, *aph(3″)-Ib*, *aph(6)-Id*, *sul2*, and *tet(A)* (Table S3).

Using IntegronFinder, ten integron clusters—comprising three complete integrons, six CALINs (clusters of *attC* sites lacking an associated integron-integrase), and one In0—were identified across the five WWTP isolates (Table S4). Specifically, four integron clusters (three CALINs and one In0) were detected in DH1_JAN2_EC13, two (two CALINs) in DH1_JAN1_EC17, one (one complete) in DH1_JAN3_EC10, two (one complete and one CALIN) in DH2_JAN2_EC21, and one (one complete) in DH2_JAN2_EC37. In contrast, a total of 32 integron clusters (10 complete, 18 CALINs, and 4 In0) were identified among 19 out of the 35 isolates of environmental origin (Table S4).

**Figure 4:**
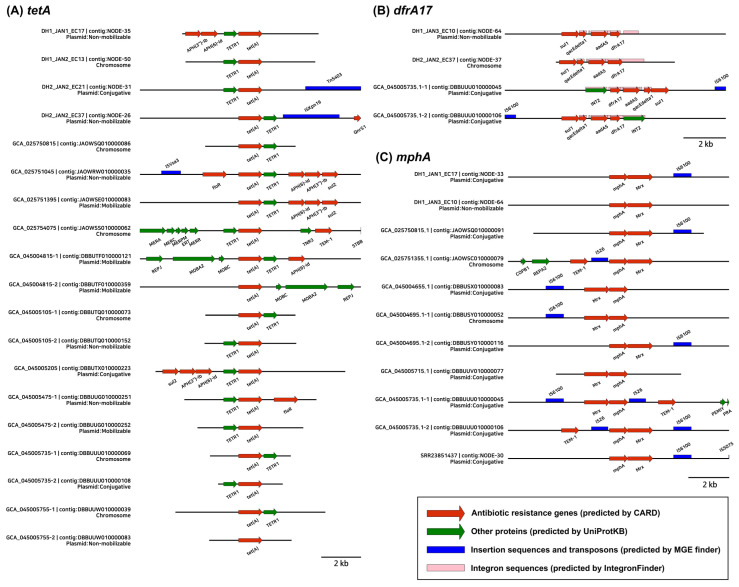
Genomic synteny and context of major antibiotic resistance genes (ARGs). The diagrams illustrate ±5-kb regions surrounding the target ARGs on contigs (≥3000 bp).

The ARG ±5 kb region synteny showed that *aph(3″)-Ib* and *aph(6)-Id* were closely located with *sul2* and *tet(A)* on plasmid contigs (Figure 4). In addition, the *tet(A)* gene was in most cases associated with the TETR1(*tetR*) gene. The *dfrA17* gene was often found as a *qacEΔ1–sul1–aadA5–dfrA17* gene cassette with complete integron sequences and was located on both chromosomal and plasmid contigs. *mph(A)* was located close to *mrx* and IS6100, and *mph(A)* linked to IS6100 was mostly located on conjugative plasmid contigs.

## 3. Discussion

In this study, WGS analysis was performed on MDR *E. coli* isolated from dewatered sludge cakes of WWTPs, and their resistomes were compared with those of 35 isolates of environmental origin. Phylogenetically, the WWTP isolates did not cluster together, but rather were dispersed in the phylogenetic tree constructed alongside environmental isolates. This result corresponds with previous studies showing WWTP-oriented *E. coli* sharing genetic diversity with clinical and environmental isolates [29,34,35].

Previous studies on antibiotic resistance in wastewater and sludge samples have relied on qPCR-based monitoring, which only detects and quantifies a limited number of targeted ARGs [23,36,37]. Although this approach is beneficial for assessing dynamic changes in the abundance of specific genes, it has several limitations: (1) the designed primers can only target specific genes; (2) determining gene variants and subtypes is difficult; and (3) demonstrating a direct link between ARGs and MGEs is challenging. In this study, using WGS, we estimated the potential localization of ARGs (chromosome or plasmid) and explored the putative associations between ARGs and MGEs. Major MGEs of clinical concern were identified in the WWTP isolates. Specifically, IncF and IncQ plasmids were found to carry ARGs and class 1 integrons linked to *qacEΔ1*–*sul1*–*aadA5*–*dfrA17* cassettes. IncF and IncI plasmids are major carriers of ARGs, including extended-spectrum beta-lactamases (ESBLs) in *Enterobacteriaceae* [38], and class 1 integrons are frequently found in environments with intense anthropogenic activity [39,40]. In addition, *mph(A)*–*mrx*–IS6100 structure was found on the conjugative plasmid contig, implying the potential rearrangement and transmission of ARGs.

In this study, some of the genotype–phenotype discrepancies that could not be resolved by the homolog model were partially explained using the variant model. For instance, the ciprofloxacin resistance in DH1_JAN1_EC17 and DH1_JAN2_EC13 could be ascribed to well-known Quinolone Resistance-Determining Region (QRDR) mutations in *GyrA* (S83L, D87N) and *ParC* (S80I), which are clinically recognized as key drivers of high-level fluoroquinolone resistance [41].

However, no such definitive evidence was found regarding fosfomycin resistance. Although resistance-associated mutations, specifically UhpT E350Q and CyaA S352T, were identified in several strains, all remained phenotypically susceptible in repeated disk diffusion assays. These findings suggest that while these amino acid substitutions may be linked to reduced susceptibility, they are insufficient on their own to confer clinical resistance.

This observation aligns with Hurwitz et al., who reported that among 46 clinical *E. coli* isolates, only the one harboring the *fosA* gene exhibited high-level resistance, despite others carrying various resistance-related variants [42]. Furthermore, Takahata et al. demonstrated that more decisive factors for resistance include the overexpression of *murA* or the loss of function in transport systems like GlpT and UhpT [43]. Collectively, our results suggest that individual substitutions such as UhpT E350Q and CyaA S352T may influence susceptibility but are not primary determinants of resistance. Consequently, further experimental validation is required to clarify their specific functional impact.

There are certain limitations to this study that should be acknowledged. First, detailed metadata for the 35 reference isolates retrieved from the NCBI Pathogen Detection database were limited to their ‘environmental’ origin; specific ecological niche data were unavailable, which may constrain the assessment of potential transmission pathways. Furthermore, while the WWTP operational conditions were documented, direct measurements of antibiotic residues or other chemical selective pressures were not performed. The absence of these data points limits our ability to correlate specific resistance profiles with precise environmental stressors.

Additionally, the use of Illumina short-read sequencing for WGS posed challenges in achieving complete plasmid or chromosomal assemblies. Consequently, the definitive classification of contigs as either chromosomal or plasmid could not be fully guaranteed. Due to these assembly limitations, investigating ARG-MGE associations was restricted. Although we attempted to analyze the ±5-kb flanking regions, several cases were excluded from the analysis because the corresponding contig lengths were insufficient. Thus, comprehensive information regarding ARG mobility remains limited, highlighting the need for future long-read sequencing (e.g., Nanopore or PacBio) to resolve these genomic structures. Finally, this study characterized only five isolates, which may not fully represent the entire diversity of multidrug-resistant (MDR) *E. coli* within the WWTP. Future studies involving a larger sample size are necessary to provide a more representative overview.

Nevertheless, the findings indicate that *E. coli* isolated from dewatered sludge cakes possesses diverse phylogenetic backgrounds and harbors MGEs and ARGs of significant clinical concern. Our results highlight the necessity of establishing management plans that account for the potential influx of ARGs into the environment during resource recovery processes, such as composting. Ultimately, this study demonstrates that WGS is an effective tool for the detailed assessment and surveillance of AMR threats in environmental matrices [44].

## 4. Materials and Methods

### 4.1. Sample Collection, Bacterial Isolation and Species Identification

Dewatered sludge cake samples were collected on 2 January 2024, from a municipal wastewater treatment plant (WWTP) located in Cheongju, Republic of Korea (36°39′ N, 127°23′ E). The facility has a treatment capacity of 280,000 m^3^/day. The treatment process involves primary sedimentation followed by the Cilium Nutrient Removal (CNR) process, an advanced biological treatment that enhances nitrogen and phosphorus removal by integrating the conventional anaerobic–anoxic–oxic (A^2^O) process with cilium-attached carriers. Following the biological process, the wastewater undergoes secondary sedimentation and UV disinfection before final discharge.

Dewatered sludge cake samples were collected from the dehydration facility of the WWTP. While both samples underwent the same digestion and dewatering processes, they were distinguished by the origin of the raw sludge: DH1 indicates the dewatered cake derived from primary sedimentation sludge, and DH2 represents the dewatered cake derived from secondary sedimentation sludge. Detailed operational parameters, including flow rate, BOD, COD, TN, TP, and total coliform counts, have been previously documented by Choi et al. (2025) [45]. After collection, samples were stored in an ice box and transported to the laboratory within 6 h for immediate analysis.

One gram of sample was mixed with 1 mL of phosphate-buffered saline, and 100 µL of the mixture was spread on mFC agar (MB Cell, Seoul, Republic of Korea). Subculturing was performed on Modified mTec agar (BD, Franklin Lakes, NJ, USA), which is a selective medium for *E. coli*. For species identification, genomic DNA was extracted using a Genomic DNA Prep Kit for Microorganisms (HiGene, Sejong-si, Republic of Korea) and amplified using the universal bacterial primer pair (27F: 5′-AGAGTTTGATCMTGGCTCAG-3′; 1492R: 5′-TACGGYTACCTTGTTACGACTT-3′). Sanger sequencing of the 16S rRNA gene was performed by Macrogen (Seoul, Republic of Korea). Sequence reads were aligned against the NCBI nucleotide database using BLASTn (v2.16.0) and the final taxonomic classification of the isolates was confirmed as *E. coli*.

### 4.2. Antibiotics Susceptibility Testing

Antibiotic susceptibility testing was performed using the disk diffusion assay. The isolates were incubated on MacConkey Agar (MB Cell, Republic of Korea) at 37 °C for 18 h. A single colony was collected, grown in Mueller–Hinton broth (MB Cell, Republic of Korea) for approximately 8 h, and diluted to an OD_600_ of 0.5 ± 0.1. A 100-μL aliquot of the diluted culture medium was spread on Mueller–Hinton Agar (MB Cell, Republic of Korea), and the following nine antibiotic disks (MB Cell, Republic of Korea) were placed on the agar: ampicillin (10 μg), cefotaxime (30 μg), ceftazidime (30 μg), gentamicin (10 μg), tetracycline (30 μg), ciprofloxacin (5 μg), chloramphenicol (30 μg), fosfomycin (includes glucose-6-phosphate; 200 μg), and nitrofurantoin (300 μg). After an 18-h incubation at 37 °C, the diameter of the inhibition zone was measured using a Vernier caliper (Fuzhou Conic Industrial Co., Ltd., Fuzhou, China). Resistance (R), intermediate resistance (I), and susceptibility (S) were determined following the cutoffs provided by the Clinical and Laboratory Standards Institute (CLSI) (Table S5).

### 4.3. Sequencing Library Preparation and WGS

Extracted DNA was quantified using a Qubit 4 fluorometer (Thermo Fisher Scientific; Waltham, MA, USA) with a Qubit dsDNA HS Assay Kit (Thermo Fisher Scientific, Waltham, MA, USA) and stored at –20 °C before use. The xGen DNA Library Prep EZ Kit (Integrated DNA Technologies, Coralville, IA, USA), HIACCUBEAD (Accugene, Incheon, Republic of Korea), and xGen UDI Primer, Index 1–16 (Integrated DNA Technologies, IA, USA) were used to prepare the sequencing library. The library was constructed according to the manufacturer’s protocol, as follows: (1) enzymatic preparation, (2) incorporation ligation, (3) post-ligation cleanup, (4) index PCR, (5) PCR cleanup, and (6) sample pooling. The pooled library was stored at −20 °C and sent to Macrogen (Seoul, Republic of Korea) for Illumina NovaSeqX sequencing.

### 4.4. WGS Data Collection from Public Database

For a comparative analysis of the five *E. coli* isolates obtained from the WWTP, additional WGS data for *E. coli* of environmental origin were collected from the NCBI pathogen database (https://www.ncbi.nlm.nih.gov/pathogens/; accessed on 1 November 2025). Strains were filtered using the following criteria: (1) organism group: *E. coli* and Shigella, (2) location: South Korea, (3) isolation source: environment, and (4) isolation type: environmental/other. In total, WGS data for 35 isolates were obtained (no detailed information on the isolation site and environment type was provided). Genome assembly data were collected where available. For samples lacking pre-assembled genomes (SRR23851437, SRR23851442, SRR23851444, and SRR23851449), raw FASTQ files were downloaded and subjected to the quality filtering process described in the following section.

### 4.5. Bioinformatics and Statistical Analysis

The adapter and low-quality sequences were trimmed using Trimmomatic (v0.39) [46]. FaQCs (v2.10) was used with default options for further quality control [47]. De novo assembly was performed using SPAdes (v4.2.0) with default settings [48] and assembly quality was assessed using BUSCO (v.6.0.0) [49]. Taxonomic classification of the isolates was performed using the GTDB-tk (v2.4.1) classify_wf workflow [50] with the GTDB release R226 reference database [51]. Open reading frames were predicted using Prodigal (v2.6.3) [52]. The predicted genes were annotated using DIAMOND BLASTp (v2.1.11) [53] against the CARD to identify ARGs [54]. Primarily, the Protein Homolog Model (v4.0.1) of CARD was used with an identity cutoff of ≥95% and a subject coverage cutoff of ≥95%. Additional analysis was performed against the Protein Variant Model (v4.0.1), with an identity cutoff of ≥90% to <100% and a subject coverage cutoff of 100%. To identify variants associated with ciprofloxacin, the protein sequences of GyrA and ParC in *E. coli* str. K-12 substr. MG1655′ were collected from NCBI, aligned using BLASTx (v2.16.0), and visualized with ESPript (v3.2) [55]. Genes were further annotated based on UniProtKB (Release 2025_03) [56] with an identity cutoff of ≥95% and a subject coverage cutoff of ≥95%.

The following tools were used to determine the potential mobility of ARGs. First, MobileElementFinder (v1.1.2) was used to identify ISs and transposons [57]. Prophage regions were predicted using DBSCAN-SWA (single version) [58] and integron sequences were identified using IntegronFinder (v2.0) [59]. Plasmid contigs were identified and typed using the mob_recon module of MOB-suite v3.0.3 [60], and replicon typing results were cross-validated using PlasmidFinder (v2.1.6) [61]. Genome synteny focusing on ARGs and adjacent MGEs was visualized using PyGenomeViz (v0.4.4) [62]. Short contigs (<3000 bp) were excluded from the synteny analysis.

Statistical differences in ARG abundance between the five WWTP isolates and 35 environmental reference strains were evaluated. Given that the data violated the assumption of normality (Shapiro–Wilk test, *p* < 0.05), the non-parametric Wilcoxon rank-sum test was performed for all comparisons. Multiple testing corrections were applied using the Benjamini–Hochberg (BH) procedure.

Multilocus sequence typing (MLST) was performed using PubMLST (https://pubmlst.org/, accessed on 5 November 2025), and the ST of each isolate was obtained using Achtman’s 7-locus MLST scheme [63]. Core-genome MLST (cgMLST) was performed using chewBBACA (v3.4.2) [64], and the scheme was downloaded from Chewie-NS (chewbbaca.online/species/10, accessed on 5 November 2025) [65]. A phylogenetic tree constructed based on the allelic profile obtained from cgMLST analysis was visualized using iTOL (v6) [66].

## 5. Conclusions

In this study, we compared the whole genomes of five *E. coli* isolates obtained from dewatered sludge cakes with 35 environmental isolates sourced from the NCBI Pathogen database. We evaluated the distribution of ARGs, their associations with MGEs, and the consistency between phenotypic and genotypic resistance. The findings revealed that *E. coli* from dewatered sludge cakes belonged to diverse sequence types (STs) and exhibited dispersed phylogenies, suggesting that WWTPs may serve as sinks for *E. coli* with diverse origins. Interestingly, despite the presence of certain known resistance-related gene variants for fosfomycin, all isolates in this study remained phenotypically susceptible, indicating that further experimental validation is required to elucidate the functional roles of these specific variants. Furthermore, the identification of clinically significant mobile ARG structures, including IncF plasmids and class 1 integrons, emphasizes the need for careful management during the recycling of dewatered sludge cakes to mitigate potential public health risks. Future studies incorporating a larger number of isolates and utilizing long-read sequencing will be essential to provide more comprehensive insights into the genomic context and potential mobility of these ARGs.

## Data Availability

The data presented in this study are openly available in [the National Center for Biotechnology Information (NCBI) Sequence Read Archive (SRA)] at [https://dataview.ncbi.nlm.nih.gov/object/PRJNA1433806?reviewer=k5np10atb7u8um702o6ld8u8l1 (accessed on 8 March 2026)], reference number [PRJNA1433806].

## Supplementary Materials

The following supporting information can be downloaded at https://www.mdpi.com/article/10.3390/antibiotics15040364/s1, Table S1: Statistical analysis of ARG abundance across drug classes according to genomic localization (Total, Chromosome, and Plasmid). The table compares 5 WWTP-derived strains and 35 reference environmental isolates. Statistical significance is shown using *p*-values and adjusted *p*-values. Table S2: Analysis of protein variants using the CARD Protein Variant Model. Observed substitutions were compared with the known catalog of resistance-associated substitutions provided by the CARD. Table S3: Characteristics of plasmid contigs. Data include contig length, potential mobility, Inc replicon/family types, and the distribution of ARGs across each contig. Table S4: Genomic characteristics of the identified integron elements. Table S5: The antibiotic disk diffusion breakpoints were determined according to the Clinical and Laboratory Standards Institute (CLSI) M100 guidelines. Figure S1: Amino acid sequence alignment of the quinolone resistance-determining regions (QRDRs) in GyrA and ParC of five *Escherichia coli* strains compared with the reference strain K-12 MG1655. Amino acid substitutions are highlighted with magenta boxes.

## Abbreviations

The following abbreviations are used in this manuscript:

AMR	antimicrobial resistance
ARB	antibiotic-resistant bacteria
ARG	antibiotic resistance gene
CARD	Comprehensive Antibiotic Resistance Database
cgMLST	core-genome multilocus sequence typing
CLSI	Clinical and Laboratory Standards Institute
ESBL	extended-spectrum beta-lactamase
HGT	horizontal gene transfer
HT-qPCR	high-throughput quantitative polymerase chain reaction
IS	insertion sequence
MDR	multidrug-resistant
MGE	mobile genetic element
PMQR	plasmid-mediated quinolone resistance
qPCR	quantitative polymerase chain reaction
QRDR	quinolone resistance-determining region
ST	sequence type
WGS	whole-genome sequencing
WWTP	municipal wastewater treatment plant
