# Supplementary figures and images for: Genomic Insight into the Mobility of Antibiotic Resistance Genes in Multidrug-Resistant Escherichia coli Isolated from Dewatered Sludge Cakes
Source: Antibiotics (Basel). 2026 Apr 1;15(4):364. doi: 10.3390/antibiotics15040364 (PMC13113784; doi:10.3390/antibiotics15040364)

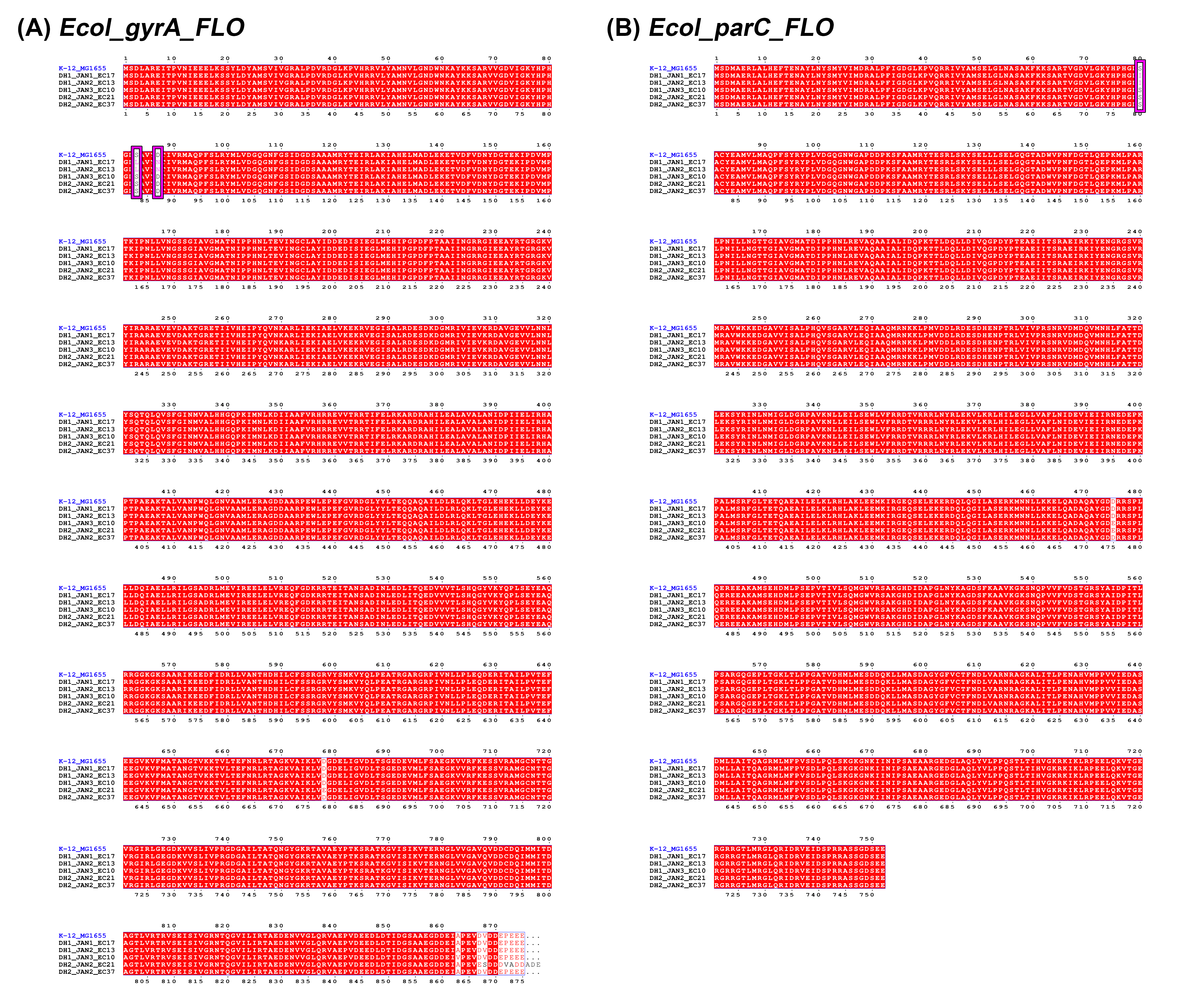

Supplement: Supplementary file 1 [file antibiotics-15-00364-s001.zip › Supplementary Figure S1.png]
